# Childhood maltreatment as a risk factor for cancer: findings from a population-based survey of Canadian adults

**DOI:** 10.1186/s12885-019-6481-8

**Published:** 2020-01-29

**Authors:** Wendy E. Hovdestad, Margot Shields, Amanda Shaw, Lil Tonmyr

**Affiliations:** Public Health Agency of Canada, Ottawa, Ontario Canada

**Keywords:** Child abuse, Child maltreatment, ACE, Adverse childhood experiences, Risk factors, Gender differences, Population health survey, Chronic disease

## Abstract

**Background:**

Childhood maltreatment (CM) is an established risk factor for various mental and substance use disorders. This study adds to existing evidence that CM may also be a risk factor for cancer.

**Methods:**

Based on data from a sample of 9783 men and 12,132 women from the 2012 Canadian Community Health Survey - Mental Health (CCHS-MH), this analysis explores mediated associations between cancer in adulthood and different levels of exposure to three types of CM—childhood physical abuse (CPA), childhood sexual abuse (CSA), and childhood exposure to intimate partner violence (CEIPV). “Cancer” was defined as an affirmative response to either of these questions: “Do you have cancer?” or “Have you ever been diagnosed with cancer?” The potential mediators were: smoking, depression, alcohol abuse/dependence, life stress, obesity, and physical activity.

**Results:**

For women, but not men, having experienced CM was significantly associated with a cancer diagnosis in adulthood, even when effects due to age and socio-demographic characteristics were controlled. Smoking, life stress, depression, and alcohol abuse/dependence reduced the strength of the association between CM and cancer in women. However, most associations remained statistically significant when controlling for effects due to these behavioural and other mediators. Evidence indicated a “dose-response” relationship, in that the likelihood of reporting cancer increased with the number of abuse types (CPA, CSA, CEIPV) reported, and with the severity of CPA.

**Conclusions:**

The analyses suggest an association between CM and cancer in women, even when the effects of known risk factors were taken into account. The association was graded, becoming stronger as CM exposure increased. Implications for the provision of cancer screening and other health care services to women with histories of CM to reduce health disparities are discussed.

## Background

Nearly half of all Canadians will develop cancer in their lifetime, and one-quarter are expected to die from the disease [1]. Cancer is the leading cause of death in Canada and the leading cause of potential years of life lost [1]. Common modifiable lifestyle risk factors include smoking [2], lack of physical activity [3], obesity [4], and heavy alcohol use [5, 6].

Recent data indicate that a third of Canadian adults had experienced at least one of three forms of childhood maltreatment (CM): physical abuse (CPA), sexual abuse (CSA), or exposure to intimate partner violence (CEIPV) [7, 8]. Moreover, childhood emotional abuse and neglect have yet to be assessed in a representative sample, so current figures may underestimate the percentage of Canadians affected by CM. Maltreatment, like other adverse childhood experiences (ACEs) (for example, family poverty, parental substance abuse) is a non-specific risk factor for long-term negative health outcomes such as psychiatric disorders and alcohol abuse [9–11], for conditions such as obesity [11–13], and for health-risk behaviours such as smoking [14].

A systematic review by Holman et al. [15] concluded that the heterogeneity of the literature limits conclusions that can be drawn, but that childhood adversity may increase cancer risk. Operationalization of ACEs in the 12 studies that they reviewed included, but was not restricted to, CM. That is, the studies used ACE data to create dichotomous variables, such that participants were scored positive or negative for exposure to each ACE [15]. In some of the studies [16], only the relation between an ACE summary score and a cancer outcome was analyzed; thus, CM was just one type of ACE that could have contributed to an increased cancer risk. Other studies in the review noted associations between cancer and specific types of CM such as physical abuse [17–19].^1^ Which different forms of CM and their frequency and severity might contribute to cancer risk is underexplored. Holman et al. suggested that to better understand the mechanisms underlying the relationship between ACEs and subsequent cancer diagnosis, future work should examine the dimensions of the ACEs in more detail, such as their frequency of occurrence in survey respondents’ childhoods. They further suggested that the interplay between ACEs and other cancer risk factors should be examined.

Mediation analyses can disentangle the interplay noted by Holman et al. by determining whether statistical relations between variables are consistent with a hypothesized pathway. In a mediating relation, the causal effect of a variable on an outcome is explained by an intervening variable [21]. For instance, in terms of tobacco use, CM predicts later smoking [22–25], which may mediate a relation between CM and cancer. However, associations between CM and cancer have been observed to persist even when effects due to smoking are statistically controlled [26]. Also, links between CM and smoking may be further complicated by associations between CM history and mental and substance use disorders [7]—people with mental and substance use disorders have higher smoking rates and are less successful at quitting [27, 28].

Similarly, associations between CM types and cancer may be mediated by alcohol use, given that heavy consumption is related to increased risk of cancer [29] and that even moderate consumption has been related to breast cancer [30]. Heavy alcohol use is itself positively related to CM history [7, 23].

Further to Holman et al.’s suggestion about exploring obesity, a meta-analysis found that CM was associated with increased odds of obesity in adulthood [13]. Obesity is a risk factor for cancer [31]. ACEs, including CM, predict physical inactivity [23, 32] which, in turn, is a risk factor for cancer diagnosis [31].

Associations between CM types and cancer may also be mediated by depression, given that at least two prospective epidemiological studies have documented that depression is a risk factor for later cancer diagnosis [33]. As well, those with ACE histories may be more sensitive to stress [34] and also may experience more stressful lives in adulthood [35]. Possible connections between life stress and increased cancer incidence have been noted [36, 37].

Given uncertainty about the nature of the interplay of risk factors noted above, this study explored associations between CPA, CSA, and CEIPV, alone and in combination, with cancer in adulthood. Based on data from the 2012 Canadian Community Health Survey - Mental Health (CCHS-MH), the analyses tested the importance to cancer of severity and frequency of each type of CM, and examined the role of cancer risk factors (smoking; depression; alcohol abuse/dependence; perceived life stress; obesity; and physical activity) as mediators in the association. Because some studies have found gender differences in associations between CM and long-term health consequences [19, 38, 39], we analysed the possibility that the association between cancer and CM differed for women and men.

## Methods

### Data and sample

The 2012 CCHS-MH was conducted by Statistics Canada using a multistage stratified clustered sampling design [40]. We have previously described our approach to analyses of these data [39, 41]. The target population was household residents aged 15 or older living in the 10 Canadian provinces. The survey excluded persons living on reserves and other Aboriginal settlements, full-time members of the Canadian Forces, and the institutionalized population. Together, these exclusions represented about 3% of the target population. The response rate was 68.9%, yielding a sample of 25,113 individuals aged 15 or older [40].

CCHS-MH respondents were asked for permission to share the information they provided with Statistics Canada’s partners, which included the Public Health Agency of Canada. Most respondents (*n* = 23,709; 94%) agreed to share. Data from the share file were used for this analysis.

The questions about CM were asked only of respondents aged 18 or older (*n* = 22,486). This study was based on data from 21,915 people (9783 men and 12,132 women); 571 records (2.5%) were excluded because of missing values.

### Measures

#### CM variables

**Fig. 1:**
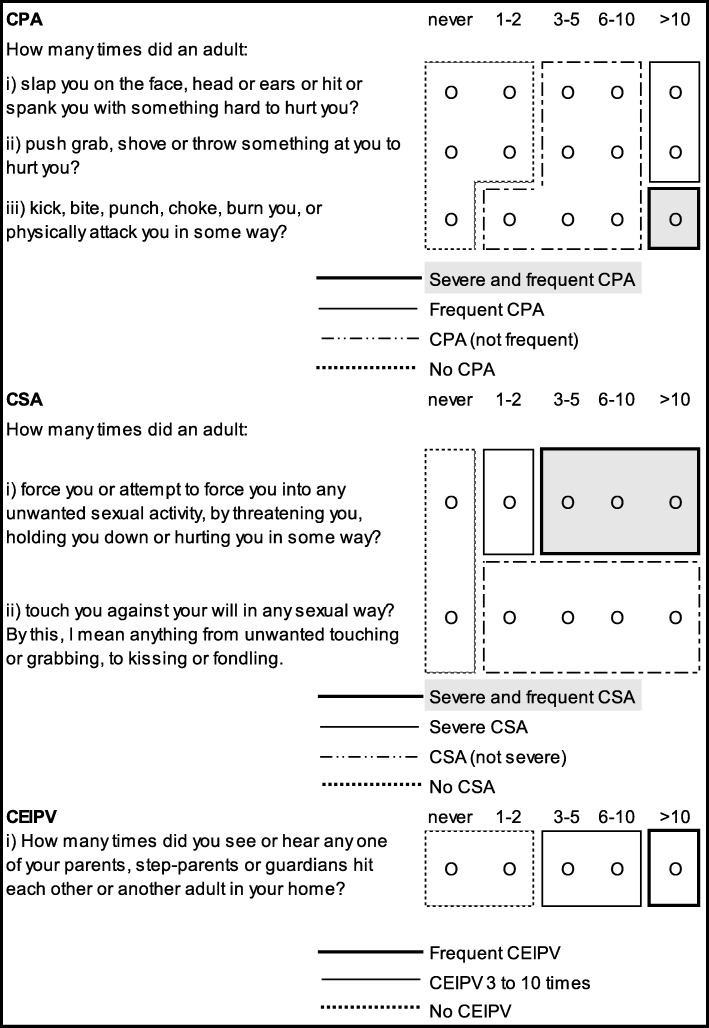
Childhood maltreatment items and definitions. Legend. CPA = Childhood physical abuse, CSA = Childhood sexual abuse, CEIPV=Childhood exposure to intimate partner violence

CPA, CSA and CEIPV were assessed with items about “*things that may have happened to you before you were 16 in your school, in your neighborhood, or in your family,*” using the items in Fig. 1.

The items for CPA and CEIPV were from the Childhood Experiences of Violence Questionnaire (CEVQ) [42]. The CSA items were similar to those used in the 2009 Canadian General Social Survey [43]. For each type of CM, binary variables were created, following CEVQ guidelines. CM severity and frequency variables were also derived (Fig. 1).

#### Cancer

To determine the presence of chronic conditions, including cancer, the CCHS-MH asked respondents about any “*long-term health conditions that have lasted or are expected to last six months or more and that have been diagnosed by a health professiona*l.” Specifically, they were asked: “*Do you have …*” each of a checklist of conditions, one of which was “*cancer,*” and “*Have you ever been diagnosed with cancer?*” Respondents who answered “*yes*” to either question were counted as having or having had cancer.

#### Mediators

A number of health risk behaviours, conditions, and comorbidities (smoking, depression, alcohol abuse/dependence, perceived life stress, obesity, and physical activity) were examined as potential mediators in the association between CM and cancer.

Smoking status was based on responses to the following:

1. At the present time do you smoke cigarettes daily, occasionally or not at all?
2. Have you ever smoked cigarettes daily?

Current daily smokers were those who answered “daily” to question 1. Former daily smokers were those who answered “occasionally” or “not at all” to question 1 and “yes” to question 2. Never daily smokers were those who responded “occasionally” or “not at all” to question 1 and “no” to question 2.

Depression and alcohol abuse/dependence (lifetime history) were assessed using the World Health Organization version of the Composite International Diagnostic Interview [44]. In addition, respondents not classified as having a lifetime history of alcohol abuse/dependence were categorized as to whether they were heavy alcohol consumers: more than 10 drinks per week for women or more than 15 drinks per week for men (either in the past year or at some point in the past) [45]. Respondents who reported having 5 or more drinks on one occasion once a week or more often in the past year were also classified as heavy alcohol consumers [45].

Perceived life stress was assessed by asking respondents if most days were not at all stressful, not very stressful, a bit stressful, quite a bit stressful, or extremely stressful.

Respondents were asked to report their height and weight. Obesity was determined using correction factors to adjust for known biases in self-reported height and weight [46]. According to cut-points recommended by the World Health Organization [47], six categories were created based on corrected BMI (kg/m^2^), ranging from underweight (less than 18.5) to obese class III (40.0 or more).

Physical activity was assessed based on the Canadian physical activity guidelines [48]. Respondents were classified as being physically active if they reported at least 150 min of moderate or vigorous physical activity in the past 7 days.

#### Socio-demographic characteristics

As in our previous work [41], the socio-demographic characteristics used as covariates in the logistic regression models were: age (continuous); marital status (married, widowed, divorced/separated, single/never married); highest level of education attained by the respondent (less than secondary graduation, secondary graduation, some postsecondary, postsecondary graduation); household income (quintiles based on household income adjusted by Statistics Canada’s low income cutoffs specific to the number of individuals in the household, the size of the community, and the survey year); immigrant status (less than 20 years in Canada; 20 or more years in Canada; Canadian-born), ethnicity (White, Black, Southeast/East Asian, off-reserve Aboriginal, other); employment status the week before the interview (employed, unemployed, not in the labour force); and place of residence (urban, rural).

### Analysis

As in our previous work [41], multivariable logistic regression analysis was used to examine associations between the CM variables and cancer, controlling for age and other socio-demographic characteristics. Regressions were run based on the binary variables for CPA, CSA, and CEIPV, and also for the CM severity and frequency variables. Where significant associations between CM and cancer were observed, the role of potential mediators (smoking status, perceived life stress, depression, alcohol use) was assessed by running five additional models, one controlling for each of the four mediating variables and a final model that included all mediators.

To test for mediation, [21] regression coefficients for the association between CM and cancer were compared before and after each mediator was added to the model (i.e., comparisons were made between each mediated model with the model controlling for only age and other socio-demographic factors). To avoid inappropriate comparison of raw regression coefficients across logistic regression models, [49] the coefficients were standardized before comparison, using a formula proposed by Menard [50]. The percentage change in the standardized regression coefficient is presented for each CM variable (i.e., change resulting from inclusion of each mediator). Attenuation of associations is evidence of mediation [21, 49, 50].

Standardized regression coefficients are useful when making comparisons of the effects of predictors across models, because all predictors are converted to a common measurement scale [50]. However, when demonstrating substantive findings for categorical variables, results based on unstandardized variables are more relevant [50]; therefore, odds ratios (ORs) are based on unstandardized regression coefficients.

We conducted all analyses with SAS Enterprise Guide 5.1 and based all estimates on weighted data. Weights, created at Statistics Canada, were used to make the data representative of the Canadian population living in the 10 provinces in 2012. The weights compensated for non-response. To account for the complex survey design of the CCHS-MH [40], variance estimates and 95% confidence intervals (CIs) were calculated using the bootstrap technique (with the SAS “proc survey” procedures).

## Results

Cancer was reported by 7.8% of women and 6.3% of men (Table 1). The most commonly reported form of CM was CPA (21.4% of women and 31.1% of men). CSA was reported by 14.4% of women and 5.9% of men, and CEIPV, by 9.0% of women and 6.7% of men.

**Table 1:** Prevalence of cancer and childhood maltreatment variables, by sex, household population aged 18 years or older, Canada, 2012

	Men	Women
	Weighted	Weighted
	percent	percent
Cancer	6.3	7.8
CPA	31.1	21.4
CPA severity and frequency		
Severe and frequent (> 10 times) CPA	2.7	2.5
Frequent (> 10 times) CPA (excluding severe and frequent CPA)	10.9	6.8
CPA (excluding frequent CPA)	17.5	12.1
No CPA	68.9	78.6
CSA	5.9	14.4
CSA severity and frequency		
Severe and frequent (> = 3 times) CSA	1.4	3.8
Severe CSA (1–2 times)	2.2	4.3
CSA (sexual touching, excluding severe CSA)	2.3	6.4
No CSA	94.1	85.6
CEIPV	6.7	9.0
Frequency of CEIPV		
More than 10 times	3.6	5.0
Three to 10 times	3.2	4.0
Never, once or twice	93.3	91.0
Co-occurrence of CPA, CSA, and CEIPV		
No abuse	65.7	69.6
1 type of abuse	26.3	19.2
2 types of abuse	6.5	8.2
3 types of abuse	1.4	3.1

Source: Statistics Canada, Canadian Community Health Survey -- Mental Health, 2012 (share file)

*CI* Confidence interval

Severe and frequent CPA was reported by 2.5% of women and 2.7% of men; severe and frequent CSA, by 3.8% of women and 1.4% of men. Frequent CEIPV was reported by 5.0% of women and 3.6% of men.

We tested for interactions between gender and CM in relation to cancer for all models. For CPA (OR = 1.9, *p* < 0.01) and CSA (OR = 1.6, *p* < 0.05) the interactions were significant but for CEIPV it was not (OR = 1.6, *p* = 0.09). All analyses were thus stratified by gender.

### Associations between CM and cancer

The prevalence of cancer by type of CM is shown in Table 2. Among men, neither the binary CM variables nor the CM severity and frequency variables were significantly associated with cancer when age or age and the other demographic variables were statistically controlled.

**Table 2:** Prevalence and adjusted odds ratios relating CPA, CSA, and CEIPV to cancer, by sex, household population aged 18 years or older, Canada, 2012

	Men			Women		
	% reporting cancer (95% CI)	Odds ratios controlling for age	Odds ratios controlling for age, and other socio-demographic factors	% reporting cancer (95% CI)	Odds ratios controlling for age	Odds ratios controlling for age, and other socio-demographic factors
		Odds (95% CI)	Odds (95% CI)		Odds (95% CI)	Odds (95% CI)
CPA						
Yes	6.0 ( 5.0, 7.1)	1.0 (0.7, 1.3)	1.0 (0.7, 1.3)	9.6 * ( 7.8, 11.4)	1.7 ** (1.3, 2.2)	1.6 ** (1.3, 2.0)
No (reference)	6.4 ( 5.5, 7.4)			7.4 ( 6.6, 8.1)		
CPA severity and frequency						
Severe and frequent (> 10 times) CPA	6.4 ( 2.5, 10.3)	1.3 (0.7, 2.7)	1.4 (0.7, 2.8)	18.4 * (10.3, 26.5)	3.6 ** (2.0, 6.5)	3.1 ** (1.7, 5.4)
Frequent (>10 times) CPA (excluding severe and frequent CPA)	6.4 ( 4.6, 8.2)	1.1 (0.8, 1.5)	1.0 (0.7, 1.5)	9.7 ( 6.0, 13.4)	1.7 * (1.1, 2.7)	1.6 * (1.0, 2.6)
CPA (excluding frequent CPA)	5.8 ( 4.4, 7.1)	0.9 (0.6, 1.2)	0.9 (0.6, 1.2)	7.8 ( 5.9, 9.7)	1.4 * (1.0, 1.9)	1.3 (1.0, 1.8)
No CPA (reference)	6.4 ( 5.5, 7.4)			7.4 ( 6.6, 8.1)		
CSA (reference no CSA)						
Yes	7.7 ( 5.2, 10.2)	1.0 (0.7, 1.5)	1.0 (0.7, 1.5)	10.8 ** ( 8.9, 12.7)	1.6 ** (1.3, 2.0)	1.4 ** (1.1, 1.8)
No (reference)	6.2 ( 5.5, 7.0)			7.3 ( 6.6, 8.1)		
CSA severity and frequency						
Severe and frequent (>= 3 times) CSA	4.8 ( 1.3, 8.3)	0.8 (0.4, 1.8)	0.8 (0.4, 1.9)	11.5 ( 6.8, 16.2)	2.0 ** (1.2, 3.3)	1.7 * (1.1, 2.7)
Severe CSA (<=2 times)	9.4 ( 3.9, 14.9)	1.3 (0.6, 2.6)	1.2 (0.6, 2.5)	11.2 * ( 7.9, 14.4)	1.7 ** (1.2, 2.5)	1.4 (1.0, 2.1)
CSA (sexual touching, excluding severe CSA)	7.8 ( 4.2, 11.4)	0.9 (0.5, 1.7)	0.9 (0.5, 1.7)	10.1 ( 7.6, 12.7)	1.4 (1.0, 1.9)	1.2 (0.9, 1.7)
No CSA (reference)	6.2 ( 5.5, 7.0)			7.3 ( 6.6, 8.1)		
CEIPV						
>=3 times	6.9 ( 4.4, 9.3)	1.3 (0.9, 2.1)	1.4 (0.9, 2.1)	10.9 * ( 7.9, 13.9)	1.9 ** (1.3, 2.6)	1.7 ** (1.2, 2.3)
<= 2 times (reference)	6.3 ( 5.6, 7.0)			7.5 ( 6.8, 8.2)		
Frequency of CEIPV						
More than 10 times	5.6 ( 2.4, 8.9)	1.2 (0.6, 2.2)	1.2 (0.6, 2.2)	10.6 ( 6.5, 14.7)	1.7 * (1.1, 2.8)	1.5 (1.0, 2.3)
Three to 10 times	8.3 ( 4.6, 12.1)	1.5 (0.9, 2.7)	1.6 (0.9, 2.8)	11.3 ( 6.9, 15.7)	2.1 ** (1.3, 3.3)	2.0 ** (1.2, 3.2)
<= 2 times (reference)	6.3 ( 5.6, 7.0)			7.5 ( 6.8, 8.2)		
Co-occurrence of CPA, CSA and CEIPV						
No abuse (reference)	6.4 ( 5.5, 7.4)			7.1 ( 6.3, 7.9)		
1 type of abuse	5.6 ( 4.5, 6.7)	0.8 (0.6, 1.1)	0.8 (0.6, 1.1)	8.3 ( 6.7, 9.8)	1.3 * (1.0, 1.7)	1.2 (1.0, 1.6)
2 types of abuse	7.6 ( 4.9, 10.3)	1.2 (0.8, 1.9)	1.2 (0.8, 1.9)	10.5 * ( 7.5, 13.6)	2.0 ** (1.4, 2.8)	1.8 ** (1.2, 2.5)
3 types of abuse	7.4 ( 1.2, 13.7)	1.4 (0.5, 3.7)	1.4 (0.5, 3.8)	13.9 * ( 8.5, 19.3)	2.7 ** (1.6, 4.5)	2.3 ** (1.4, 3.7)

Source: Statistics Canada, Canadian Community Health Survey -- Mental Health, 2012 (share file)

*CPA* Childhood physical abuse, *CSA* Childhood sexual abuse, *CEIPV* Childhood exposure to intimate partner violence, *CI* onfidence interval

* *p* <0.05 ** *p* <0.01 Significantly different from reference

Among women (Table 2), the binary CPA, CSA, and CEIPV variables were significantly associated with cancer when controlling for age and the other socio-demographic variables. When severity and frequency of CPA and CSA were considered, a dose-response relationship emerged for both; the pattern was less clear for CEIPV. When the additive impact of one, two, or three types of CM was considered, a dose-response relationship was also noted.

Preliminary logistic regression analyses revealed that smoking status, perceived life stress, lifetime history of depression, and alcohol abuse/dependence were associated as expected with cancer (Table 3). However, obesity was not related to cancer, and the relation between physical activity and cancer was the reverse of what was expected—those who were more physically active were more likely to report cancer [OR = 1.3 (CI 1.0–1.6)]. Thus, neither obesity nor physical activity was included in the models.

**Table 3:** Prevalence and adjusted odds ratios relating selected variables (potential mediators) to cancer, female household population aged 18 years or older, Canada, 2012

	% reporting cancer (95% CI)	Odds ratios controlling for age, other socio-demographic factors and:						
		Obesity Odds (95% CI)	Smoking status Odds (95% CI)	Physical activity level Odds (95% CI)	Perceived life stress Odds (95% CI)	Life-time history of depression Odds (95% CI)	Alcohol abuse/dependence Odds (95% CI)	All mediators Odds (95% CI)
Obesity - BMI category (range kg/m2)								
Underweight (less than 18.5)	11.1 ( 5.1, 17.0)	1.7 (0.9, 3.5)						1.6 (0.8, 2.9)
Normal weight (18.6 to 24.9) (reference)	6.6 ( 5.5, 7.7)							
Overweight (25.0 to 29.9)	8.9 ** (7.6, 10.1)	1.1 (0.8, 1.4)						1.1 (0.8, 1.4)
Obese Class I (30.0 to 34.9)	8.1 ( 6.3, 9.8)	0.9 (0.7, 1.2)						0.9 (0.7, 1.2)
Obese Class II (35.0 to 39.9)	9.1 ( 6.3, 12.0)	1.1 (0.7, 1.7)						1.1 (0.8, 1.7)
Obese Class III (40.0 or more)	7.6 ( 4.6, 10.7)	1.0 (0.6, 1.6)						1.0 (0.6, 1.6)
		1.4 (0.7, 2.7)						
Smoking status								
Daily smoker	9.8 ** ( 7.7, 12.0)		1.7** (1.3, 2.3)					1.5** (1.2, 2.0)
Former daily smoker	10.9 ** ( 9.3, 12.6)		1.3* (1.1, 1.6)					1.3* (1.0, 1.6)
Never a daily smoker (reference)	6.3 ( 5.5, 7.0)							
Physically active								
Yes	8.6 ( 7.3, 9.9)			1.3* (1.0, 1.6)				1.3* (1.0, 1.7)
No (reference)	7.4 ( 6.5, 8.2)							
Perceived life stress								
Not at all stressful (reference)	10.3 ( 8.0, 12.6)							
Not very stressful	7.0 * ( 5.9, 8.1)				1.0 (0.7, 1.3)			1.0 (0.7, 1.3)
A bit stressful	7.4 * ( 6.3, 8.6)				1.4* (1.0, 1.8)			1.3* (1.0, 1.8)
Quite a bit stressful	7.5 ( 5.7, 9.2)				1.6* (1.1, 2.3)			1.5* (1.0, 2.2)
Extremely stressful	12.5 ( 6.6, 18.4)				2.8** (1.5, 5.1)			2.5** (1.4, 4.7)
Life-time history of depression (reference no history of depression)								
Yes	9.3 ( 7.2, 11.5)					1.5** (1.1, 2.0)		1.3 (1.0, 1.8)
No (reference)	7.6 ( 6.9, 8.3)							
Alcohol use								
Life-time history of alcohol abuse/dependence	8.7 ( 6.5, 10.9)						1.6** (1.2, 2.2)	1.4* (1.0, 1.9)
No history of abuse/dependence but above low-risk guidelines	8.6 ( 6.3, 11.0)						1.4* (1.0, 2.0)	1.3 (0.9, 1.8)
No history of abuse/dependence and within low-risk guidelines (reference)	7.6 ( 6.8, 8.4)							

Source: Statistics Canada, Canadian Community Health Survey -- Mental Health, 2012 (share file)

* *p*<0.05 ** *p*<0.01 Significantly different from reference

### Effects of mediating variables on CM-cancer association for women

When smoking status, perceived life stress, lifetime history of depression, and alcohol abuse/dependence were included in the models along with age and the other socio-demographic variables, the binary CPA and CEIPV variables continued to be associated with cancer for women, as shown in Table 4. As well, evidence suggested that the dose-response relationship persisted for analyses of number of co-occurring types of CM, and for severity and frequency of CPA. The association of CSA with cancer was not statistically significant in the fully mediated models, and the association with cancer was significant for CEIPV 3 to 10 times, but not for more than 10 times.

**Table 4:** Adjusted odds ratios relating CPA, CSA, and CEIPV to cancer, female household population aged 18 years or older, Canada, 2012

	Odds ratios controlling for age, other socio-demographic factors and:										
	Odds (95% CI)	smoking status		perceived life stress		depression		alcohol abuse/dependence		all mediators	
		Odds (95% CI)	%∆^a^	Odds (95% CI)	%∆^a^	Odds (95% CI)	%∆^a^	Odds (95% CI)	%∆^a^	Odds (95% CI)	%∆^a^
CPA (reference no CPA)	1.6 ** (1.3, 2.0)	1.6 ** (1.2, 2.0)	-7	1.5 ** (1.2, 1.9)	-12	1.5 ** (1.2, 2.0)	-8	1.6 ** (1.2, 2.0)	-6	1.4 ** (1.1, 1.8)	-26
CPA severity and frequency (reference no CPA)											
Severe and frequent (> 10 times) CPA	3.1 ** (1.7, 5.4)	2.7 ** (1.6, 4.8)	-11	2.7 ** (1.5, 4.8)	-13	2.9 ** (1.7, 5.1)	-2	2.9 ** (1.7, 5.1)	-3	2.3 ** (1.3, 4.1)	-25
Frequent (>10 times) CPA (excluding severe and frequent CPA)	1.6 * (1.0, 2.6)	1.6 * (1.0, 2.5)	-3	1.5 (1.0, 2.4)	-13	1.6 * (1.0, 2.4)	-10	1.6 * (1.0, 2.5)	-6	1.4 (0.9, 2.2)	-25
CPA (excluding frequent CPA)	1.3 (1.0, 1.8)	1.3 (0.9, 1.7)		1.3 (0.9, 1.7)		1.3 (0.9, 1.7)		1.3 (0.9, 1.7)		1.2 (0.9, 1.6)	
CSA (reference no CSA)	1.4 ** (1.1, 1.8)	1.3 * (1.1, 1.7)	-14	1.3 * (1.1, 1.7)	-14	1.3 * (1.0, 1.7)	-17	1.4 ** (1.1, 1.7)	-10	1.2 (1.0, 1.5)	-44
CSA severity and frequency (reference no CSA)											
Severe and frequent (>= 3 times) CSA	1.7 * (1.1, 2.7)	1.6 (1.0, 2.5)	-15	1.5 (0.9, 2.5)	-21	1.6 (1.0, 2.5)	-16	1.6 * (1.0, 2.5)	-12	1.3 (0.8, 2.1)	-49
Severe CSA (<=2 times)	1.4 (1.0, 2.1)	1.4 (0.9, 2.0)		1.4 (0.9, 2.0)		1.4 (0.9, 2.0)		1.4 (1.0, 2.0)		1.2 (0.8, 1.8)	
CSA (sexual touching, excluding severe CSA)	1.2 (0.9, 1.7)	1.2 (0.9, 1.7)		1.2 (0.9, 1.7)		1.2 (0.8, 1.6)		1.2 (0.9, 1.7)		1.1 (0.8, 1.6)	
CEIPV (reference no CEIPV (<= 2 times))	1.7 ** (1.2, 2.3)	1.6 ** (1.2, 2.2)	-10	1.5 * (1.1, 2.1)	-21	1.6 ** (1.2, 2.2)	-8	1.6 ** (1.2, 2.3)	-4	1.4 * (1.0, 1.9)	-35
Frequency of CEIPV (reference no CEIPV (<= 2 times))											
More than 10 times	1.5 (1.0, 2.3)	1.4 (0.9, 2.1)		1.3 (0.8, 2.0)		1.4 (0.9, 2.1)		1.4 (1.0, 2.1)		1.2 (0.8, 1.8)	
Three to 10 times	2.0 ** (1.2, 3.2)	1.9 * (1.2, 3.1)	-6	1.8 * (1.1, 2.8)	-14	1.9 ** (1.2, 3.1)	-5	2.0 ** (1.2, 3.2)	-2	1.7 * (1.1, 2.7)	-22
Co-occurrence of CPA, CSA and CEIPV (reference no abuse)											
1 type of abuse	1.2 (1.0, 1.6)	1.2 (0.9, 1.5)		1.2 (0.9, 1.5)		1.2 (0.9, 1.5)		1.2 (0.9, 1.6)		1.1 (0.9, 1.4)	
2 types of abuse	1.8 ** (1.2, 2.5)	1.7 ** (1.2, 2.5)	-6	1.6 ** (1.1, 2.3)	-15	1.7 ** (1.2, 2.5)	-8	1.7 ** (1.2, 2.5)	-3	1.5 * (1.1, 2.2)	-26
3 types of abuse	2.3 ** (1.4, 3.7)	2.1 ** (1.3, 3.4)	-11	2.0 ** (1.2, 3.4)	-14	2.1 ** (1.3, 3.4)	-9	2.2 ** (1.4, 3.4)	-7	1.7 * (1.1, 2.8)	-33

Source: Statistics Canada, Canadian Community Health Survey -- Mental Health, 2012 (share file)

Note: The odds ratios are based on unstandardized regression coefficients

*CPA* Childhood physical abuse, *CSA* Childhood sexual abuse, *CEIPV* Childhood exposure to intimate partner violence, *CI* confidence interval

^a^%∆=percent change: Refers to the percent change in the standardized regression coefficient for child maltreatment variable resulting from the inclusion of mediating variables in the logistic regression model compared with the model only controlling for socio-demographic factors (indicated if childhood maltreatment variable was significant in the model controlling for socio-demographic factors only)

*p <0.05 **p <0.01

## Discussion

As recommended by Holman et al. [15], our study of three types of CM as risk factors for later cancer, using a Canada-representative data set, helps to disentangle the interplay between ACEs and cancer risk. We found a positive relationship between CM and cancer for women, but not men. When smoking, life stress, depression, and problems with alcohol were included in the models along with age and the other socio-demographic variables, CPA and CEIPV continued to be associated with cancer for women, although the relationships were attenuated. As well, a dose-response relationship (more CM, more cancer) persisted in analyses of number of co-occurring types of CM, and for severity and frequency of CPA.

The association between women’s greater experience of CM and their increased cancer risk is consistent with Coker et al.’s [51] findings based on a sample of nearly 5000 American women. Coker et al. observed that women who had experienced CSA were more likely than those who had not to self-report cervical cancer. Further, they found that women’s risk of cervical cancer increased as their lifetime exposure to violence increased from zero to three types. Our findings are also similar to earlier research in which the association between cancer incidence (as assessed by hospital discharge records) and ACEs (including CM) was partly, but not fully, attenuated by demographics, smoking behaviour, and other variables [52]. However, most of the work in this area has examined dose-response relationships between cancer and ACEs in general, rather than CM in particular [32, 53–55].

To some extent, the gender differences documented in this study may reflect patterns in the incidence and prevalence of prostate and breast cancer, which account for about 25% of all cancers in Canadian men and women, respectively. Prostate cancer is typically diagnosed in men at age 65 or older [56]. Therefore, an association between CM and cancer may not have been apparent among men, most of whom had not reached the age at which prostate cancer has developed and advanced to the stage of detectability. A similar observation was made by Korpimäki et al. [57], who noted that the working-age sample used in their study might have been too young to detect cancers in men.

Among the strengths of the present work is that the data are representative of the Canadian adult population, among whom few comparable studies have been conducted. The measures of three types of CM were of high quality. As well, it was possible to conduct analyses at the total number of types of CM experienced, the frequency of EIPV, and the frequency and severity of CPA and CSA. Measures of many potential mediators were also available for analysis.

Despite these strengths, the findings should be considered in the context of several limitations which, taken together, require that this study should be seen as an early exploration of the interplay between CM, potential mediators, and cancer risk. This is a retrospective cross-sectional study; it cannot be used to make conclusions about causality, although mediation analyses tested a hypothesized causal chain. Regarding the measures used, our work suggests that associations between CM and cancer vary by type of CM and thus an important limitation of our work is the unavailability of measures of childhood neglect and childhood emotional abuse, as well as of other ACEs.

Height and weight (from which obesity was derived) and physical activity were self-reported and might have been more accurate had they been based on direct measurements.

The non-specific nature of a self-reported diagnosis of cancer is sub-optimal,^2^ although Brown et al. [58] found that self-reported data on cancer have acceptable agreement with medical chart information (also see [59]). As well, the self-reported estimate of cancer prevalence is higher than a similar estimate derived from a national cancer institute [1]^3^; Brown et al. [58] noted a similar inflation using self-report data. Further, highly virulent cancers that may be associated with CM may be under-reported here due to early morbidity and mortality. The inability to detect mortality from cancer is another limitation of the self-reported data.

Inflammation was not available in this dataset, and so it could not be examined as a potential mediator of the associations between CM and cancer. Inflammation is a biological process that has been studied in relation to CM and tumor growth. Based on their systematic review, Coelho et al. [60] concluded that CM experiences may cause enduring physiological responses that are detrimental to immune system functioning and result in a chronic inflammatory state.

As well as past stressors, inflammation can be triggered by “proinflammatory” diets, which, in turn, are associated with common types of cancers [61, 62]. Inflammation related to past stressors and to poor diet may both be ways that childhood experiences have lifelong impacts, in that children with more severe experiences of maltreatment may, compared to those not maltreated or those who had milder maltreatment experiences, lack appropriate care including provision of nourishing food and teaching of good nutrition habits.

Kerr et al. [31] noted that obesity is a key contributory factor associated with cancer risk and mortality, and that childhood diet is a risk factor for cancer.

## Conclusions

Analyses of data from the Canadian Community Health Survey - Mental Health revealed a statistically significant relationship between CM and cancer for women, but not for men.

The associations between women’s experiences of CSA and cancer were mediated by socio-demographic characteristics and cancer risk factors. However, for CPA and CEIPV, the associations remained statistically significant under mediation; smoking, perceived life stress, and other psychosocial factors did not substantially alter the link. The findings suggest a “dose-response” relationship between CPA severity and frequency and cancer among women. Women’s experience of more types of CM was also related to increasing risk.

The evidence from the present paper suggesting that health behaviours may not be the only pathway by which CM contributes to cancer in adulthood has implications for future analyses and for practice.

A broad perspective on ACE-relevant absences of childhood care, as well as the presence of traumatic stress, could be useful in future research intended to address health inequalities related to cancer [63].

Some individuals at risk of cancer may need specifically targeted health care interventions, due to their CM histories, in order to ensure equality of health outcomes. Along these lines, using linked population-based primary and secondary care data, Woodhead et al. [64] found that women who had serious mental illnesses and were known to secondary mental health services were less likely than other women in the same population to have been screened for breast or cervical cancer. Woodhead et al. did not assess CM, but it is possible that the relation of CM history to mental illness and to psychological and life circumstances would impair the uptake of health-relevant advice and also complicate the provision of services to people with histories of CM.

Policies intended to reduce cancer health disparities might usefully take note of the prevalence of CM in populations most at risk for cancer. Cancer health disparities related to social disadvantage may manifest via reduced levels of screening, follow-up, and treatment because of patient characteristics and health care provider factors [65]. Alcalá et al. [66] found that the association between ACEs and cancer screening was complex. Future longitudinal research with better measures of cancer might usefully explore the ways that CM might function as a risk factor for later cancer differentially in subpopulations that differ in terms of social disadvantage.

We suggest that CM histories should be considered as a patient characteristic that might hamper meaningful engagement with health care systems, even in Canada where preventive medical care is relatively accessible. More broadly, because CM is a nonspecific risk factor for a multitude of negative physical health and mental health outcomes, and for health and mental health risk behaviours (e.g., poor diet, substance abuse, involvement in violent interpersonal relationships), CM prevention should continue to be a priority for public health.

## Data Availability

The data are available for analysis from Statistics Canada.

## Abbreviations

ACES: Adverse childhood experiences
BMI: Body mass index
CCHS-MH: Canadian Community Health Survey – Mental Health
CEIPV: Childhood exposure to intimate partner violence
CEVQ: Childhood Experiences of Violence Questionnaire
CI: Confidence interval
CM: Childhood maltreatment
CPA: Childhood physical abuse
CSA: Childhood sexual abuse
ORs: Odds ratios

## Footnotes

1 Also see Hughes’ review of CM and other ACES and their moderate effects on increased smoking and cancer risk [20].

2 Cancer is a group of many diverse diseases characterized by abnormal cell growth; the non-specific self-reported cancer measure in this health survey dataset is sub-optimal.

3 In 2009 2.4% of the Canadian population had been diagnosed with cancer in the past 10 years.
